# The Fate of Phosphate: Assessing Dietary Intake and Urinary Excretion in Swedish Adolescents

**DOI:** 10.1016/j.cdnut.2024.103799

**Published:** 2024-06-19

**Authors:** Fredrik Söderlund, Jennifer Gransten, Emma Patterson, Anna Karin Lindroos, Sanna Lignell, Carolina Donat-Vargas, Linnea Bärebring, Susanna C Larsson, Maria Kippler, Agneta Åkesson

**Affiliations:** 1 Unit of Cardiovascular and Nutritional Epidemiology, Institute of Environmental Medicine, Karolinska Institutet, Stockholm, Sweden; 2 Department of Risk and Benefit Assessment, Swedish Food Agency, Uppsala, Sweden; 3 Department of Internal Medicine and Clinical Nutrition, Institute of Medicine, Sahlgrenska Academy, University of Gothenburg, Gothenburg, Sweden; 4 ISGlobal, Barcelona, Spain; 5 Department of Preventive Medicine and Public Health, School of Medicine, Universidad Autónoma de Madrid-IdiPaz, CIBERESP (CIBER of Epidemiology and Public Health), Madrid, Spain; 6 Department of Surgical Sciences, Medical Epidemiology, Uppsala University, Uppsala, Sweden; 7 Unit of Metals and Health, Institute of Environmental Medicine, Karolinska Institutet, Stockholm, Sweden

**Keywords:** dietary phosphate, phosphate additives, urinary phosphorus, adolescents, 24-h recalls, ICP-MS

## Abstract

**Background:**

A high total phosphorus (P) intake has been proposed to promote endothelial dysfunction and atherosclerosis. A diet rich in foods containing P additives could contribute to an excessive intake, potentially reflected as increased concentration of P in urine.

**Objectives:**

This study aimed to assess the intake of total dietary P, P additives, and its sources and examine their correlation with urinary P in a cross-sectional national study in Swedish adolescents.

**Methods:**

We constructed a database of P additives and applied it to the foods consumed by 3099 participants in the representative school-based dietary survey Riksmaten Adolescents 2016–17. Intake of total dietary P and P additives were assessed using two 24-h recalls. Urinary P was analyzed in a subsample of 756 participants using inductively coupled plasma mass spectrometry. Spearman rank correlation (ρ) was used to assess the association between dietary P intake and urinary P excretion.

**Results:**

The mean (SD) intake of total P was 1538 (±667) mg/d. Food containing P additives were consumed by 92% of adolescents and the median (IQR) intake was 49 (22–97; range: 0.01–947) mg/d, corresponding to 5% (1%–6%; range: 0%–50%) of total P. The main contributing food to P additives was cola drinks, while the main contributing food group was sausage dishes. Total P intake was weakly correlated with urinary P (ρ = 0.12; *P* < 0.01) but not with intake of P additives.

**Conclusions:**

Nearly, all participants consumed P additives, contributing to an average of 5% of total P intake but ranging up to 50%. The intake of total P, but not P additives, was weakly reflected in the urinary P. Access to more comprehensive information on P additives in foods would improve further evaluation of potential health consequences.

## Introduction

Phosphorus (P) is essential for all living organisms, being involved in many processes throughout the body. P metabolism is tightly regulated and involves complex interactions among the gut, bones, and kidneys [1]. Absorption of dietary P in the duodenum and jejunum in the small intestines stimulates the release of parathyroid hormone from the parathyroid glands and fibroblast growth factor 23 from osteocytes and osteoblasts in bone, which reduces P reabsorption in the renal tubule and increases its renal clearance [2,3]. The intake of P should, therefore, under normal circumstances, be reflected in the excretion of urinary P (U-P) [2].

Dietary P is mainly found in its natural form as organic P in protein-rich foods, such as meat, fish, dairy products, and legumes [3]. Another form, inorganic P, is commonly found as an additive in various foods, as it is added during the manufacturing processes to provide functional advantages in food such as preservation, texture improvement, emulsification, leavening agent, or flavor enhancement [4]. Inorganic P has been reported to have high bioavailability and is nearly fully absorbed in the intestines [5,6], unlike organic P, which has an absorption rate of roughly 65%, depending on P source [3,7]. Excessive consumption of P, especially in the form of additives, has raised health concerns, with a suggested link between acute P loading and endothelial dysfunction, as well as habitual intake and atherosclerosis [8,9]. The upper limit for total dietary P intake was set to 3000 mg/d in the Nordic Nutrition Recommendations 2023, but no recommendations were made for P additives [10].

The quantity of inorganic P intake is largely unknown. There are currently no food labeling requirements for P additives, and as food composition databases only represent total P (i.e., not differentiated by natural and added P), data on P additives are limited and imprecise. Thus, with few exceptions, previous estimations of dietary P intake have disregarded P additives [11]. However, estimations of the most common P additives, conducted by the European Food Safety Authority (EFSA), have indicated that inorganic P may, on average, contribute to 20%–30% of total P intake [12]. Moreover, foodstuffs containing P additives are common, found in more than a third of foodstuffs in Finland [13], and about half in the United States [14] and Australia [15]. The aim of this study was to assess the intake of total dietary P, P additives, their sources, and their correlation with U-P in a representative cross-sectional study among Swedish adolescents.

## Methods

### Study design and population

**FIGURE 1:**
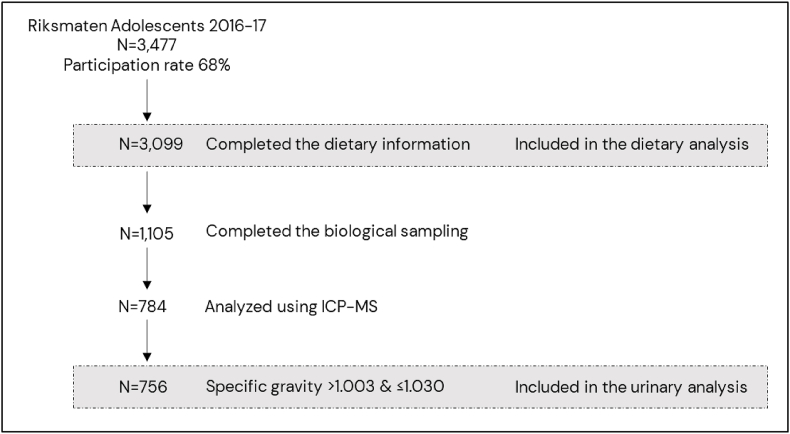
Flow chart of the study population from Riksmaten Adolescents 2016–17. ICP-MS, inductively coupled plasma mass spectrometry.

To determine intake of dietary P, we used data from Riksmaten Adolescents 2016–17, a school-based dietary survey from a nationally representative selection of Swedish children and adolescents in school grades 5, 8, and 11 (mean ages 12, 15, and 18 y, respectively), conducted by the Swedish Food Agency (SFA) as described previously [16]. In brief, schools in Sweden were randomly invited to participate based on municipality characteristics, geographical spread, and size of the school, with 1 or 2 classes included from each school. About 40% of the included schools were randomly selected for additional blood and spot urine sampling. Trained staff visited the schools to instruct participants on how to conduct the 24-h recalls, collected urine samples, and measured height and weight. The school visits were similar for all grades, but additional time was added in grade 5 visits. Reported energy intake <800 and >3500 kcal/d were manually checked for irregularities and removed if considered implausible, leaving 3099 adolescents with dietary information for 2 d, with 1105 completed the biological sampling, which was collected on the day of the first 24-h recall (Figure 1). Of these, 789 urine samples were available for P analysis in this project, of which 4 samples were excluded owing to insufficient urine volume, and 1 was excluded because of error in the chemical analysis, resulting in 784 urine samples analyzed for U-P. The Regional Ethical Board in Uppsala approved the study (No. 2015/190), and written informed consent was obtained from participants and their legal guardians in the biomonitoring part of the study, while opt out consent was applied for the remaining participants.

### Dietary assessment

Dietary intake was assessed using the method RiksmatenFlexDiet, a biomarker-validated, web-based dietary assessment method developed for Riksmaten Adolescents 2016–17, as previously described in detail [17]. Participants registered types of foods and beverages along with the quantities consumed, for 2 nonconsecutive days, including a weekend day. Portion sizes were specified using a picture portion guide. The first self-administered 24-h recall was conducted during the visit of the study staff. The second 24-h recall was randomly assigned 2–7 d after the first recall. To simplify for the participants, the assessment used both generic and specific foods, with 778 prespecified foods and composite dishes. During the registration, the participants could, in a second step, add additional information about types of meat, fish, vegetarian alternatives, and so on, for further clarification. The energy and nutrient intakes were calculated automatically by linking to the SFA food composition database, version Riksmaten Adolescents 2016–17.

### Total phosphorus and phosphorus additives

To estimate total dietary P intake, the values of P in the food composition database were updated to the most available information on P content in foods from SFA as per 23 January, 2024. The P content of foods had been either determined through direct nutrient analysis (42%) or calculated from the included constituents (58%).

The P additives assessed are presented in Table 1. Additional P additives such as various distarch phosphates (E 1412-1414) were not included owing to insufficient information regarding their usage levels. To create the P additive database, total P from the food composition database was combined with the industry information on P additives obtained by EFSA [12].

**TABLE 1:** Additives included in the phosphate database.

E-number	Common name
E338	Phosphoric acid
E339(i–iii)	Mono-, di-, and trisodium phosphate
E340(i–iii)	Mono-, di-, and tripotassium phosphate
E341(i–iii)	Mono-, di-, and tricalcium phosphate
E343(i–ii)	Mono- and dimagnesium phosphate
E450(i–iii, v–vii, ix)	Di-, tri-, and tetrasodium diphosphate, tetrapotassium diphosphate, dicalcium diphosphate, calcium dihydrogen diphosphate, magnesium dihydrogen diphosphate
E451(i)	Pentasodium triphosphate, pentapotassium triphosphate
E452(i–iv)	Sodium polyphosphate, potassium polyphosphate, sodium calcium polyphosphate, calcium polyphosphate

In a first step, all food items were assigned into categories according to Annex II of the Regulation (EC) No 1333/2008 on food additives [18]. Food categories not approved for P additive use were classified as free from P additives, while food items in food categories classified as potentially containing P additives were further checked for P additives in the ingredient lists. This was accomplished in the following manner: for single-ingredient food items, we checked ingredient lists of ≥15 randomly selected (organic or conventional) products available on websites of common grocery stores on the Swedish market (e.g., ICA Gruppen, Coop Sverige AB, and Axfood), covering ∼90% of the Swedish market in 2023. The websites were checked by JG and verified by FS. If <15 products were available, all products were checked. If ≥10% of products checked contained P additives, the food was classified as containing P additives and further explored in the second step. An exception was made if only the available products containing P additives were gluten-free products (considered less commonly consumed), containing baking powder as sole P additive, in which the food was classified as free from P additives. Food items from fast-food restaurant chains were checked for ingredients containing P additives on their respective websites and classified accordingly.

In a second step, we added the industry data on P additives, provided in mean added P_2_O_5_ (in milligrams per kilogram or milligrams per liter as appropriate), and converted to P from the molar mass according to P = P_2_O_5_ × 0.4365. Industry data on P additives were available for 89 authorized uses within 60 food categories [defined according to Annex II of the Regulation (EC) No 1333/2008 on food additives]. Additional information on the industry data are presented in Supplemental Table 1. In 10 of 778 food items, including highly processed ingredients like processed cheese, we used the industry-provided estimates for P additive levels instead of the prior calculated P from the national food composition database, as the former was considered most updated (Supplemental Table 2). The content in composite dishes was estimated based on the ingredients with P additives, using common recipes, which concerned 52 dishes. In the last step, we combined the P additives database with the dietary intake data from RiksmatenFlexDiet, provided in grams per food.

### Assessment of urinary phosphorus

Urine samples (stored in −80 °C) were thawed overnight and diluted 1:10 in nitric acid, and U-P was analyzed at Karolinska Institutet using inductively coupled plasma mass spectrometry (ICP-MS; Agilent 7900; Agilent Technologies). The overall mean limit of detection was <0.026 mg/L, and no sample had a U-P concentration below this limit of detection. As quality control samples, 2 commercial reference materials were included in every run (Seronorm Trace Element urine 1403080 L1 and Seronorm Trace Element urine 1706878 L-2), and there was a good agreement between recommend and obtained P values (approximate value: 809 mg/L compared with obtained mean ± SD: 750 ± 61 mg/L; *n* = 20 and approximate value: 349 mg/L compared with obtained mean ± SD: 337 ± 15 mg/L; *n* = 21). U-P was adjusted for the overall mean specific gravity (SG) of the group of 1.021 according to U-P × (1.021 − 1)/measured SG − 1. SG was measured using a digital refractometer (EUROMEX RD712; Clinical Refractometer). Participants with SG <1.003 and ≥1.030 representing unrealistic values (too diluted or too concentrated; *n* = 28) were excluded, leaving 756 participants in the analyses based on urine concentrations.

### Statistical methods

The assessment of total P and P additive intake was appraised based on the 778 available food items. The mean intake of total P and the median intake of P additives were calculated based on data from the two 24-h recalls. BMI was defined according to international references, accounting for sex and exact age for participants aged 18 y or younger [19]. The correlation between the intake of total P and P additives (milligrams per day) with SG-adjusted U-P (milligrams per liter) was assessed using Spearman rank correlation (ρ), in all and restricted to those participants who consumed P additives (>0 mg/d). Statistical tests were 2 sided with a significance level set to 0.05, performed using STATA/BE (version 17.0; Stata Corporation) and presented as mean and SD or median and IQR.

## Results

Altogether, 16% (*n* = 125 food items) of all single-ingredient food items reported in the dietary recalls (from 37 food groups; 34%) were estimated to contain P additives. The estimated mean of P added to food items was 479 mg/kg, and the food items with the highest estimated P content were processed cheese containing emulsifying salts (10,535 mg/kg), chewing gum (4986 mg/kg), milkshake with extra protein (4000 mg/kg), and seafood (mainly from frozen products) (4000 mg/kg).

**FIGURE 2:**
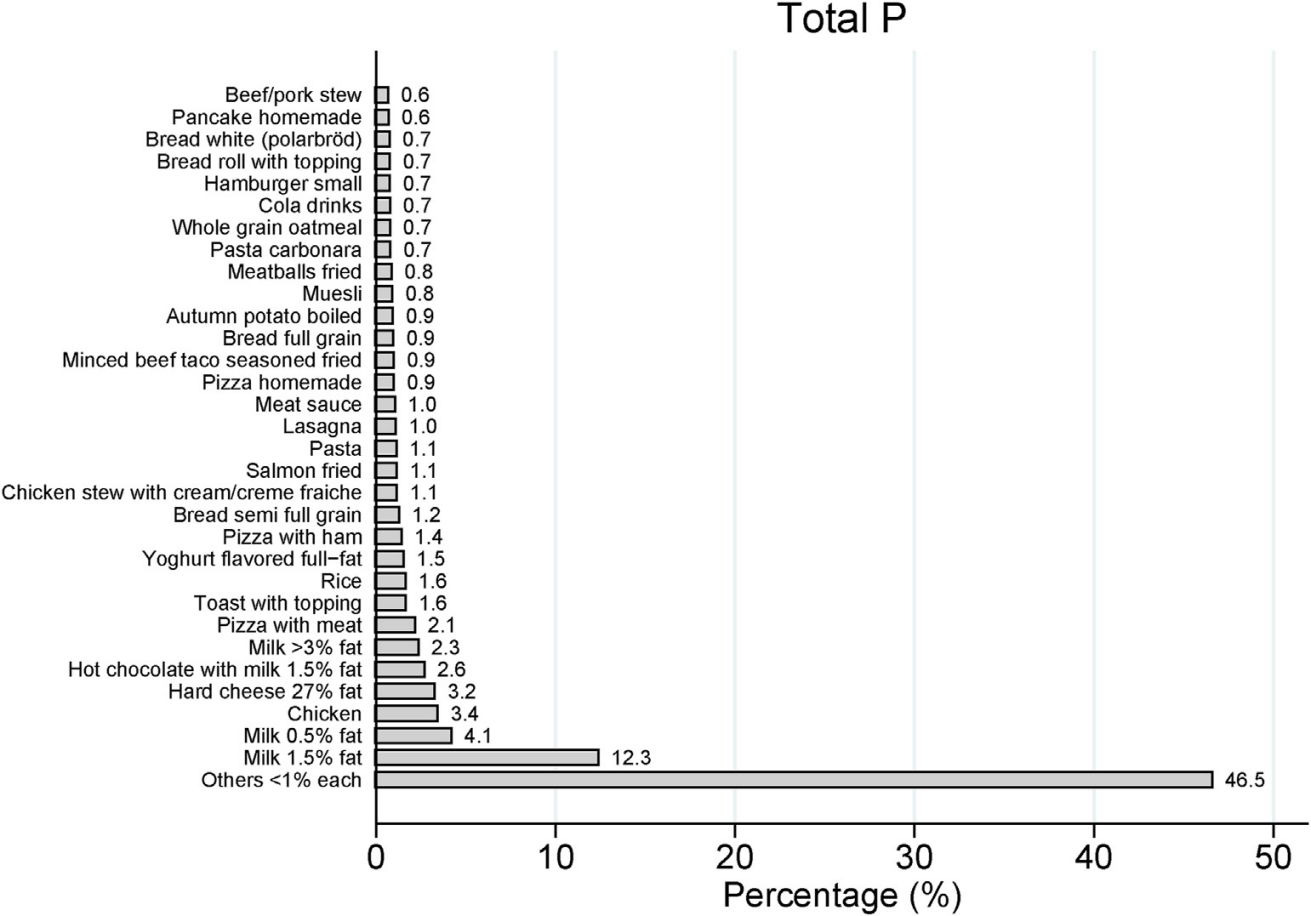
Sources of total phosphate and their respective contributions to intake of total phosphate in the whole study population (*N* = 3099).

The characteristics and dietary P intake of the whole study population and the subsample with urine samples are presented in TABLE 2, TABLE 3, respectively. Among the 3099 adolescents, intake of total P ranged from 91 to 7141 mg/d with the mean of 1538 (SD 667) mg/d. Moreover, 4% (*n* = 111) of adolescents had an intake of total P that exceeded 3000 mg/d, and 1% (*n* = 16) had an intake that exceeded 4000 mg/d. The main single-ingredient food items contributing most to total P intake were milk 1.5% fat (12%), milk 0.5% fat (4%), and pizza (4%) (Figure 2). The main food groups contributing with the highest mean intake of total P were milk, hard cheese, and milk drink, chocolate drizzle, milkshake, and smoothie with yogurt (Table 4).

**TABLE 2:** Major characteristics and dietary intake of the whole study population, Riksmaten Adolescents 2016–17 (*N* = 3099)^1^.

Characteristics^2^	All (*N* = 3099)	5th grade (*n* = 1049)	8th grade (*n* = 1050)	11th grade (*n* = 1000)
Age (y) (min; max)^3^	15 (10; 21)	12 (10; 13)	15 (13; 17)	18 (16; 21)
Sex				
Female (%)	1710 (55)	559 (53)	574 (55)	577 (58)
Male (%)	1389 (45)	490 (47)	476 (45)	423 (42)
BMI (kg/m^2^)^4^	20.8 (3.8)	18.9 (3.3)	20.7 (3.3)	22.9 (3.7)
Overweight/obesity (%)^4^^,^^5^	21	22	17	24
Dietary intake				
Energy (kJ/d)	8900 (3500)	8100 (2900)	9300 (3800)	9500 (3600)
Energy (kcal/d)	2100 (800)	1900 (700)	2200 (900)	2300 (900)
Protein (g/d)	88 (40)	81 (31)	91 (41)	93 (45)
Carbohydrate (g/d)	241 (101)	223 (85)	252 (114)	249 (100)
Fat (g/d)	85 (40)	76 (35)	88 (43)	92 (40)
Total P (mg/d)	1538 (667)	1440 (562)	1603 (731)	1573 (687)
Added P (mg/d)^6^	43 (16–91)	35 (12–75)	44 (17–92)	56 (21–106)
Added P (mg/d)^6^^,^^7^	49 (22–97)	40 (17–82)	50 (23–96)	62 (27–111)
Added P (mg/kg BW/d)^6^^,^^7^	0.8 (0.3–1.6)	0.8 (0.3–1.7)	0.8 (0.30–1.6)	0.8 (0.3–1.6)

Abbreviations: BW, body weight.

1 Dietary intake was calculated as an average of two 24-h recalls.

2 Continuous variables are presented as mean ± SD. Categorical variables are presented as percentages.

3 Based on 3098 adolescents, as 1 individual had a missing variable for age.

4 Excluding individuals with missing variables for weight and height; all, *N* = 3073; 5th grade, *n* = 1031; 8th grade, *n* = 1047; 11th grade, *n* = 995.

5 Defined according to International Obesity Task Force for participants aged 18 y or younger and according to BMI ≥25 and ≥30 for participants older than 18 y.

6 Median (IQR).

7 Only including individuals consuming added P; all, *N* = 2837; 5th grade, *n* = 947; 8th grade, *n* = 966; 11th grade, *n* = 924.

**TABLE 3:** Major characteristics and dietary intake of the subsample with urine samples analyzed for phosphorus, Riksmaten Adolescents 2016–17 (*n* = 756)^1^.

Characteristics^2^	Subsample (*n* = 756)	5th grade (*n* = 215)	8th grade (*n* = 287)	11th grade (*n* = 254)
Age (y) (min; max)	15 (11; 21)	12 (11; 13)	15 (14; 16)	18 (17; 21)
Sex				
Female (%)	418 (55)	104 (48)	160 (56)	154 (61)
Male (%)	338 (45)	111 (52)	127 (44)	100 (39)
BMI (kg/m^2^)^3^	21.0 (3.7)	18.9 (2.9)	20.6 (3.2)	23.2 (3.8)
Overweight/obesity (%)	22	22	17	27
Dietary intake				
Energy (kJ/d)	9300 (3500)	8400 (2900)	9800 (3800)	9600 (3400)
Energy (kcal/d)	2200 (800)	2000 (700)	2300 (900)	2300 (800)
Protein (g/d)	91 (38)	86 (32)	96 (43)	91 (35)
Carbohydrate (g/d)	249 (99)	224 (80)	266 (111)	251 (96)
Fat (g/d)	90 (42)	81 (38)	95 (44)	94 (41)
Total P (mg/d)	1609 (653)	1528 (561)	1720 (752)	1550 (586)
Added P (mg/d)^4^	48 (18–96)	43 (11–86)	45 (20–96)	58 (23–103)
Added P (mg/d)^4^^,^^5^	53 (24–98)	46 (18–92)	50 (25–104)	62 (29–109)
Added P (mg/kg BW/d)^4^^,^^5^	1.0 (0.4–1.8)	1.1 (0.4–2.0)	0.9 (0.4–1.8)	0.9 (0.5–1.6)
Urinary phosphorus^6^ (mg/L)	611 (297)	624 (259)	599 (267)	613 (354)

Abbreviations: BW, body weight.

1 Dietary intake was calculated as an average of two 24-h recalls.

2 Continuous variables are presented as mean ± SD. Categorical variables are presented as percentages.

4 Median (IQR).

5 Only including individuals consuming added P; subsample, *n* = 695; 5th grade, *n* = 196; 8th grade, *n* = 266; 11th grade, *n* = 233.

6 Adjusted for specific gravity to 1021 g/mL

3 Defined according to International Obesity Task Force for participants aged 18 y or younger and according to BMI ≥25 and ≥30 for participants older than 18 y.

**TABLE 4:** Average intake of total phosphate and phosphate additives per food group over the 2 d in the whole population (*N* = 3099).

Food group	Total phosphate (mg)		Added P (mg)		
	Mean ± SD	Range	Median (IQR)	Mean ± SD	Range
Algae products	3 ± 0	3–3	0 (0–0)	0 ± 0	0–0
Beer	3639 ± 3260	443–6960	0 (0–0)	0 ± 0	0–0
Berries; fresh or frozen	914 ± 863	68–2366	0 (0–0)	0 ± 0	0–0
Blood products/dishes	3361 ± 0	3361–3361	0 (0–0)	0 ± 0	0–0
Brawn	14 ± 0	14–14	0 (0–0)	0 ± 0	0–0
Broth	38 ± 0	38–38	0 (0–0)	0 ± 0	0–0
Buns, cookies, cakes, etc.	4672 ± 4510	83–16,035	726 (0–4410)	1707 ± 2153	0–6612
Butter	59 ± 84	0–119	0 (0–0)	0 ± 0	0–0
Candy containing chocolate	15,320 ± 21,217	342–52,767	0 (0–0)	0 ± 0	0–0
Candy, nonchocolate	266 ± 330	33–644	s0 (0–0)	0 ± 0	0–0
Cereals—breakfast cereals	14,596 ± 22,461	232–81,018	0 (0–0)	0 ± 0	0–0
Cheese dishes	4656 ± 6460	88–9224	0 (0–0)	0 ± 0	0–0
Cheese with vegetable fat	744 ± 702	89–1484	0 (0–56)	19 ± 32	0–56
Chewing gum	112 ± 0	112–112	6103 (6103–6103)	6103 ± 0	6103–6103
Chocolate	22,278 ± 15,167	7687–43,377	0 (0–0)	0 ± 0	0–0
Cider, alcopops, drinks	585 ± 863	0–2071	0 (0–0)	0 ± 0	0–0
Cocoa products	792 ± 0	792–792	0 (0–0)	0 ± 0	0–0
Coffee	7147 ± 0	7147–7147	0 (0–0)	0 ± 0	0–0
Coffee, tea; with milk	10,068 ± 10,844	2400–17,736	292 (0–629)	292 ± 239	123–461
Crackers	763 ± 342	372–1007	0 (0–0)	0 ± 0	0–0
Cream cheese and quark	6963 ± 6977	18–22,560	0 (0–0)	0 ± 0	0–0
Cream, *creme fraiche*	5026 ± 4140	102–10,581	0 (0–0)	0 ± 0	0–0
Crisp bread	11,806 ± 18,554	21–44,572	0 (0–0)	0 ± 0	0–0
Dessert cheese	1963 ± 2055	638–4330	0 (0–0)	0 ± 0	0–0
Desserts	1182 ± 1472	6–4755	0 (0–0)	111 ± 373	0–1453
Egg	29,924 ± 24,504	1835–46,922	0 (0–0)	0 ± 0	0–0
Egg products and dishes	14,014 ± 14,804	650–29,927	0 (0–0)	0 ± 0	0–0
Fish and seafood	4512 ± 1930	3147–5877	1597 (0–4791)	1597 ± 2259	0–3194
Fish and seafood; products and dishes	6499 ± 12,847	10–53,107	0 (0–0)	505 ± 2421	0–12,358
Fish: fresh, frozen, or cooked	17,089 ± 20,698	180–46,002	0 (0–0)	0 ± 0	0–0
Fish: fried unbreaded	52,768 ± 72,807	1286–104,251	0 (0–0)	0 ± 0	0–0
Fish: smoked	9482 ± 0	9482–9482	0 (0–0)	0 ± 0	0–0
Flavored sour milk, yogurt	44,358 ± 64,750	2898–140,985	0 (0–0)	0 ± 0	0–0
Flour, starch, bran	2871 ± 3423	55–6682	0 (0–0)	0 ± 0	0–0
Food grains, cereals	4292 ± 5957	240–14,742	0 (0–0)	0 ± 0	0–0
Fruit and berries: canned	72 ± 31	50–94	0 (0–0)	0 ± 0	0–0
Fruit and nut mixes: bars	1772 ± 796	1209–2335	0 (0–0)	0 ± 0	0–0
Fruit: fresh frozen	2651 ± 5377	41–25,739	0 (0–0)	30 ± 145	0–711
Fruits and berries: dried	338 ± 665	0–1825	0 (0–0)	0 ± 0	0–0
Fruit juice, etc.	5958 ± 11,035	9–33,344	0 (0–0)	0 ± 0	0–0
Gruel	1850 ± 0	1850–1850	0 (0–0)	0 ± 0	0–0
Hamburger with bread (meat, fish, poultry, vegetarian)	29,555 ± 23,796	7489–67,320	0 (0–55)	1366 ± 3318	0–8139
Hard cheese	110,077 ± 169,535	6412–305,724	0 (0–0)	0 ± 0	0–0
Ice cream	2381 ± 3160	57–13,391	0 (0–0)	6 ± 18	0–64
Jam, marmalade, jelly, applesauce, etc.	735 ± 887	8–2171	0 (0–0)	0 ± 0	0–0
Juice, soda, cider without alcohol	8039 ± 22,600	0–68,281	0 (0–0)	7587 ± 22,760	0–68,281
Legumes (beans, lentils, and peas)	3654 ± 4545	246–14,048	0 (0–0)	0 ± 0	0–0
Liqueur	0 ± 0	0–0	0 (0–0)	0 ± 0	0–0
Mayonnaise-based salad, blends	1588 ± 1716	8–4789	0 (0–0)	0 ± 0	0–0
Meat products, meat dishes	20,125 ± 29,579	246–95,404	0 (0–0)	540 ± 1925	0–9346
Meat: fresh, frozen, cooked	18,330 ± 19,076	235–53,765	0 (0–0)	0 ± 0	0–0
Meat: processed	6903 ± 9110	23–28,459	71 (0–1192)	616 ± 968	0–2657
Milk	257,453 ± 432,058	108–1,174,580	0 (0–14)	777 ± 2044	0–5413
Milk drink, chocolate drizzle, milkshake, and smoothie with yogurt	66,533 ± 104,710	4346–250,804	0 (0–0)	32 ± 71	0–159
Mixed liquid edible fat	0 ± 0	0–0	0 (0–0)	0 ± 0	0–0
Mixed solid edible fats	2225 ± 3853	0–6674	0 (0–0)	0 ± 0	0–0
Mushroom	373 ± 238	102–680	0 (0–0)	0 ± 0	0–0
Mustard, ketchup, HP sauce, soy, “seasoning”	1394 ± 2142	0–6273	0 (0–0)	5 ± 14	0–40
Natural sour milk, yogurt	20,791 ± 23,894	228–56,145	0 (0–0)	0 ± 0	0–0
Nuts, seeds	4264 ± 4299	216–13,411	0 (0–0)	0 ± 0	0–0
Offal, organs; products and dishes	754 ± 715	113–1760	0 (0–0)	0 ± 0	0–0
Oil	0 ± 0	0–0	0 (0–0)	0 ± 0	0–0
Other fats (lard, tallow, coconut oil)	0 ± 0	0–0	0 (0–0)	0 ± 0	0–0
Other sweetened beverages, water-chocolate	1703 ± 2409	0–3407	0 (0–0)	0 ± 0	0–0
Pancakes, waffles, crepes	22,315 ± 26,796	424–60,358	9 (0–2245)	1118 ± 2224	0–4453
Pasta	50,802 ± 46,777	13,513–103,287	0 (0–0)	0 ± 0	0–0
Pasta dishes	25,422 ± 33,369	1170–99,579	0 (0–0)	535 ± 1514	0–5034
Pizza, pie, pirogue, ready-to-eat sandwich	21,980 ± 42,046	89–201,856	32 (0–831)	701 ± 1418	0–6753
Porridge	20,863 ± 24,581	1363–69,712	0 (0–0)	0 ± 0	0–0
Potato chips, popcorn, etc.	10,835 ± 18,688	45–57,881	0 (0–0)	0 ± 0	0–0
Potato products, potato dishes	7511 ± 11,817	137–39,486	0 (0–0)	305 ± 1047	0–4074
Potatoes	24,975 ± 28,478	738–83,568	0 (0–0)	3029 ± 9581	0–31,887
Poultry	81,678 ± 159,717	534–321,238	0 (0–304)	152 ± 304	0–608
Poultry products, poultry dishes	24,521 ± 36,176	492–105,131	0 (0–267)	2010 ± 5474	0–15,549
Processed cheese	10,933 ± 0	10,933–10,933	4097 (4097–4097)	4097 ± 0	4097–4097
Rice	1373 ± 580	678–2218	0 (0–0)	0 ± 0	0–0
Rice cakes	3444 ± 0	3444–3444	0 (0–0)	0 ± 0	0–0
Rice, rice noodles	38,764 ± 74,510	452–150,514	0 (0–0)	0 ± 0	0–0
Roe, caviar	1538 ± 1859	224–2853	0 (0–0)	0 ± 0	0–0
Root vegetables	1274 ± 2562	5–9566	0 (0–0)	34 ± 126	0–472
Salad, mixed food	3905 ± 5212	57–18,336	0 (0–66)	181 ± 372	0–1203
Sauce, dressing, mayonnaise	4755 ± 9230	22–37,800	0 (0–0)	367 ± 1196	0–6170
Sausage	14,026 ± 18,451	74–57,347	0 (0–1279)	1297 ± 2394	0–7146
Sausage dishes	27,648 ± 25,367	9711–45,585	8999 (0–23,058)	8999 ± 9941	1970–16,029
Seafood, squid: fresh, frozen, cooked	6168 ± 0	6168–6168	0 (0–0)	0 ± 0	0–0
Soft bread	27,473 ± 33,459	414–116,090	0 (0–0)	2547 ± 6175	0–22,847
Soft drinks: nonenergy	4381 ± 7310	0–12,820	0 (0–12,820)	4273 ± 7401	0–12,820
Soup	9182 ± 10,025	291–27,454	0 (0–158)	407 ± 1055	0–3369
Sour milk, yogurt, and acidified products	11,816 ± 3554	9302–14,329	0 (0–0)	0 ± 0	0–0
Soy protein, wheat protein, Quorn; products and dishes	3633 ± 3216	58–8321	0 (0–0)	46 ± 165	0–596
Spices	268 ± 0	268–268	0 (0–0)	0 ± 0	0–0
Spirits	0 ± 0	0–0	0 (0–0)	0 ± 0	0–0
Sports drinks, energy drinks	9383 ± 0	9383–9383	0 (0–0)	0 ± 0	0–0
Sugar, syrup, honey	40 ± 76	0–153	0 (0–0)	0 ± 0	0–0
Sugar-free candy	3 ± 0	3–3	0 (0–0)	0 ± 0	0–0
Supplements	5532 ± 5954	1243–15,076	0 (0–70)	62 ± 105	0–242
Sweet soups, fruit puree, and dessert sauce	954 ± 1165	18–3483	0 (0–0)	0 ± 0	0–0
Taco shells	3172 ± 0	3172–3172	0 (0–0)	0 ± 0	0–0
Tea	2571 ± 0	2571–2571	0 (0–0)	0 ± 0	0–0
Vegetable juice, root vegetable juice	291 ± 204	57–434	0 (0–0)	0 ± 0	0–0
Vegetable mixtures with root vegetables and/or legumes	2181 ± 697	1590–2950	0 (0–0)	0 ± 0	0–0
Vegetable products and milk substitutes	14,446 ± 17,898	1790–27,102	4078 (0–9934)	4078 ± 4141	1149–7006
Vegetable, root vegetable, and legume dishes and products	1553 ± 2119	12–7712	0 (0–0)	228 ± 821	0–4296
Vegetables	2338 ± 3843	13–16,198	0 (0–0)	27 ± 181	0–1216
Water, mineral water	0 ± 0	0–0	0 (0–0)	0 ± 0	0–0
Whey butter, whey cheese (*mesvaror*)	809 ± 27	790–828	0 (0–0)	0 ± 0	0–0
Wine	397 ± 350	11–787	0 (0–0)	0 ± 0	0–0

**FIGURE 3:**
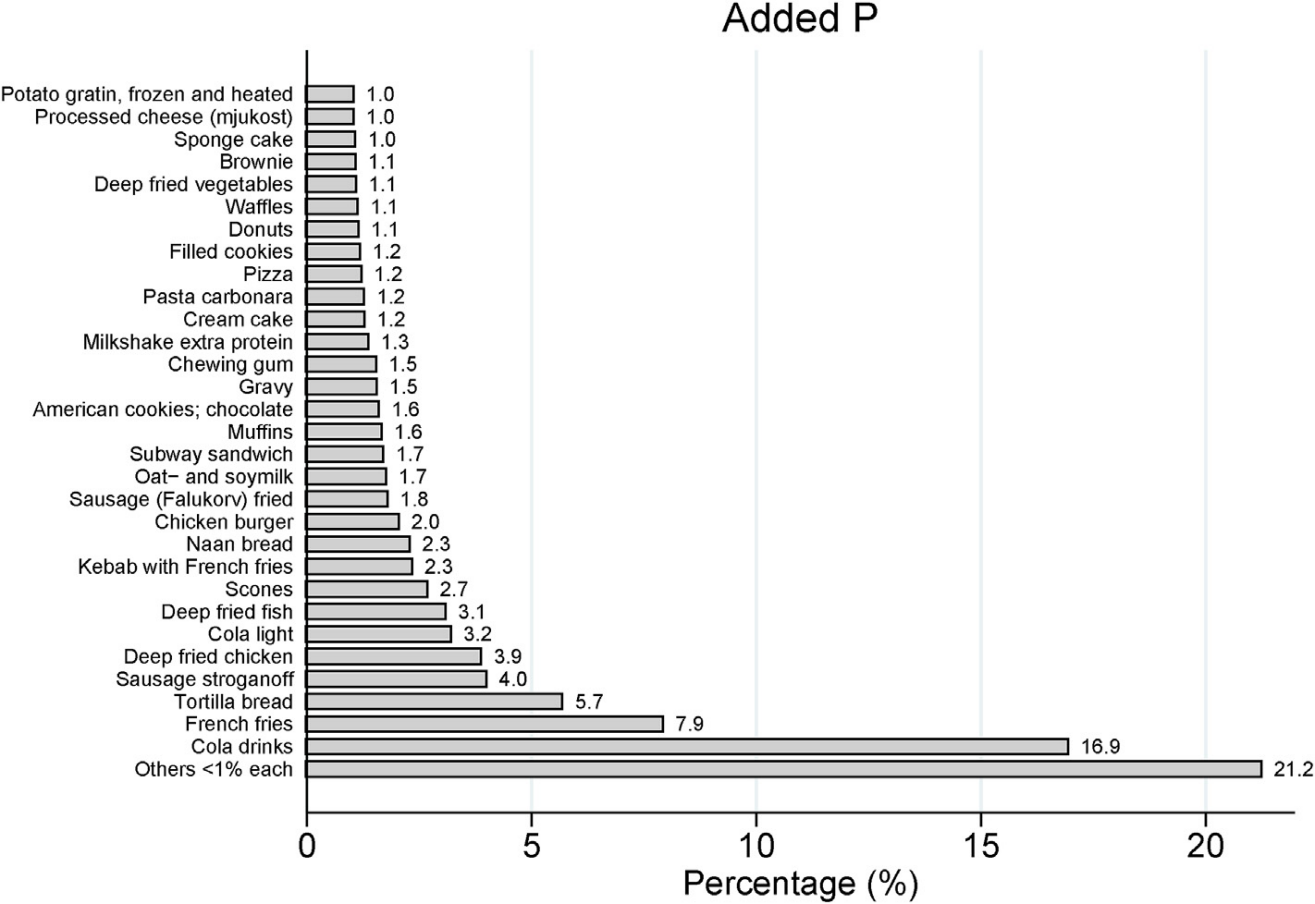
Sources of phosphate additives and their respective contributions to intake of phosphate additives in the whole study population (*N* = 3099).

A total of 2837 (92%) adolescents consumed food items containing P additives, ranging from 0.01 to 947 mg/d (median: 49 mg/d; IQR: 22–97 mg/d) (Table 2), corresponding to 0.8 mg/kg body weight/d. P additives contributed, on average, to 5% (1%–6%; range: 0%–50%) of total P. The median intake of P additives ranged from 35 mg/d in the youngest age group to 56 mg/d in the oldest (Table 2). The main single-ingredient food items contributing to intake of P additives were cola drinks (17%), French fries (8%), and white tortilla bread (6%) (Figure 3). The main food groups contributing to the highest median intake of P additives were sausage dishes, followed by juice, soda, nonalcoholic cider, and chewing gum (Table 4).

In the subsample with U-P (*n* = 756), intake of total P and P additives was overall similar to that of the whole group (Table 3). A total of 695 (92%) adolescents consumed food items containing P additives, and their intake ranged from 0.05 to 583 mg/d (median: 53 mg/d; IQR: 24–98 mg/d). The main contributing food items to total and P additive intake were similar to the whole population (Supplemental Figures 1 and 2). The median intake of P additives ranged from 43 mg/d in the youngest age group to 58 mg/d in the oldest (Table 3).

The levels of SG-adjusted U-P found in the subsample ranged from 11 to 3109 mg/L, with the mean 611 (SD: 297) mg/L (Table 3). The univariate Spearman correlation (ρ) between the intake of total P and SG-adjusted U-P was 0.12 (*P* < 0.01). The correlation varied slightly between the age groups (ρ = 0.07, 0.22, and 0.05 in the 5th, 8th, and 11th grade students, respectively). Restricting the analysis to the first 24-h recall only had no impact on the correlation (ρ = 0.12; *P* < 0.001). No correlation was observed between P additives and U-P in any of the analyses. In participants with a total P intake of >3000 mg total P/d, the mean U-P concentration was 665 (SD: 254) mg/L, while in those with ≤3000 mg total P/d the corresponding U-P was 609 (SD: 298) mg/L (*P* = 0.3).

## Discussion

For this cross-sectional study, we developed a database containing P additives, which was used to assess the intake of total P and P additives among Swedish adolescents. More than 90% of adolescents consumed P additives during the two 24-h recalls, and 4% reported an intake exceeding the tolerable upper intake level for total P intake of 3000 mg/d set in the Nordic Nutrition Recommendations 2023 [10]; however, it should be noted that the tolerable upper intake level concerns the habitual intake, while this study covered 2 days only with a likely wider distribution of the intake. Total P intake was only weakly reflected in the urinary excretion of P. The food group contributing to the highest intake of P additives was sausage dishes, while the largest single food contributor was cola drinks.

In healthy individuals, the main route of urinary P excretion is through the kidneys, where nearly 100% is filtered in the glomeruli, and ∼80%–90% are then reabsorbed in the proximal tubules [7]. Oral P loading may disrupt the P homeostasis through increased production of fibroblast growth factor 23 and parathyroid hormone secretion [20,21], which, in turn, regulates U-P excretion [22]. The production of these hormones also decreases intestinal P absorption and reabsorption in the kidneys through reduction of 1,25-dihydroxyvitamin D_3_ [23], responsible for the activity of the sodium-dependent phosphate transporters 2a and 2c [7]. Elevated levels of fibroblast growth factor 23 have been proposed to increase endothelial dysfunction and cardiovascular events [[24], [25], [26]]. As P additives, mainly in the form of inorganic P, are absorbed to a greater extent in the intestines compared to organic P [3,5,6], intake of P additives, especially during P loading, might have the potential to increase cardiovascular disease risk. Based on data from the French NutriNet-Santé cohort, an association was observed between the intake of one of the assessed P additives, trisodium phosphate (E339), and subsequent increased risk of coronary artery disease (hazard ratio: 1.06; 95% CI: 1.00, 1.12) [27]. Another recent study found associations of higher U-P with increased risk for composite cardiovascular disease and myocardial infarction [28]. However, no association [29], or even a protective association [30], with higher U-P concentrations has been reported.

### Intake of total P and P additives

The mean total P intake was in line with previous reports [12]. The intake of P additives (median 43 mg/d or 0.8 mg/kg body weight/d) was, however, lower than previously estimated in other populations [12,27,31,32]. In the NutriNet-Santé cohort, mean intake of P additives in adults was 357 mg/d and 80% of the participants consumed P additives [27]. In Japanese children (1–6 y), the intake of P additives was 11.2 mg/kg body weight/d [31], while in Polish children (<18 y) and young Polish adults (18–24 y), the corresponding intake of P additives from meat products alone was 5.5 and 2.2 mg/kg body weight/d, respectively [32].

EFSA conducted an evaluation aiming to capture different exposure scenarios to P additives in 2019 [12]. In 33 dietary surveys from 19 different European countries, the estimated mean intake among adolescents ranged between 74 and 1945 mg/d (126–1867 mg/d for adults) in the different surveys, depending on the scenario. In this study, we used the industry-reported mean content of P additives in those foods with the greatest number of food samples provided to better reflect what these adolescents likely encountered, than using the maximum permitted levels of P additives. This approach may be more conservative than that used by EFSA. Consequently, in EFSA’s most conservative approach (the non–brand-loyal scenario), the estimated mean intake of P additive ranged between 74 and 298 mg/d in the different surveys.

### Correlation between P intake and U-P

The correlation between intake of total P and U-P in this study (ρ = 0.12) was comparable with that reported in a previous observational study involving a Swedish population of healthy older women (ρ = 0.1), which assessed the first morning void U-P and the intake of total P from food frequency questionnaires, however, lacking sufficient information on P additives [28]. Randomized controlled crossover trials, comparing a low with a high P additive diet, have observed an approximate 25% decrease in 24-h U-P and a similar subsequent increase, following a low and a high P additive diet [33,34], with fibroblast growth factor 23 following a similar pattern. Another randomized controlled trial assessed acute effects of P intake from 4 different sources (meat, whole grains, cheese, and supplements), compared with those of a control diet [35]. All diets provided 500 mg/d of dietary P, and the experimental diets had an additional 1000 mg/d from the specific P source. A significant increase in 24-h U-P was observed when the major source of P intake was from supplements or meat, compared with both the controls and the other food groups. These results demonstrate that an acute response to the intake of P (total and additives) is reflected in urine. A possible explanation to the weak correlation observed in this study is reduced absorption of P following a chronic high intake, where less P is absorbed, and remnant levels are found in the stool [23]. However, this cannot be assessed with this study design.

### P additives in foodstuffs

The estimated amount of P additives in single-ingredient food items in this study (16%) was lower than that previously reported in foodstuffs in Finland (36%), United States (44%), and Australia (44%), indicating a potential underestimation in the results [[13], [14], [15]]. The Finnish study, assessing the prevalence of P additives in the Finnish food supply, rather than the intake, found that all food categories contained inorganic P additives and that all products in the food categories of processed and cream cheese, cola and energy drinks, and processed meats contained inorganic P additives [13]. Conversely, in the NutriNet-Santé cohort, assessing P additives from emulsifiers in an adult population, found that ≥50% of total P intake came from cakes and biscuits, while <5% and <1% came from unsweetened and sweetened soft drinks, respectively [27]. The latter results differ from those of this study, where P additives from cola drinks were the major source of intake, which is likely due to a higher consumption of soft drinks in adolescents than in adults [36].

### Assessing P additives

P additives are authorized for 108 different uses (corresponding to 65 food categories) within the European Union. The industry data provided included 89 authorized uses (corresponding to 60 food categories), indicating a data gap in 19 authorized uses. Furthermore, as chemical analysis of foodstuffs has trouble differentiating between additives and naturally occurring P, they are an unreliable source of information, and further development of analytical tools is needed [12]. Therefore, future studies need reliable data on individual foodstuffs, either from chemical analysis or from industry usage, to improve the understanding of P additives in food.

### Strengths and limitations

The main strength of this study is the use of industry data to assess the intake of P additives. Another strength is the study population, consisting of a representative sample of adolescents in Sweden with detailed information on consumption of food and beverages. This study also has limitations. We assessed the intake of P additives from food items where industry usage levels were reported. However, it is unclear whether the data reflect the usage levels for all products or producers. Furthermore, the method used for assessing dietary intake (RiksmatenFlexDiet) was limited to 778 food items and dishes and may not have been granular enough to capture all foods consumed containing P additives, and although we used validated dietary recalls for 2 nonconsecutive days, we could not rule out the influence of recall bias and unmeasured day-to-day variation in seldom consumed food items. Additionally, we lack information on contribution of other P additives than those listed in Table 1, indicating that actual exposure might be higher owing to unaccounted sources. This highlights the need for comprehensive assessments of aggregated sources of P additives to elucidate overall P exposure. The lack of correlation between intake of P additives and U-P may stem from various sources. Estimating dietary intake is challenging, and the dietary recalls might not have adequately captured total P or P additive intake. Likewise, a single spot urine sample might not adequately reflect the daily P excretion, especially when the sampling was not based on first voided morning urine. The use of 24-h urine collection is considered better for measurement of urinary P excretion [12]. Nevertheless, this might not be feasible in larger populations, such as the current one.

In conclusion, although dietary intake of P additives was lower than previously reported, nearly all participants consumed P additives contributing to an average of 5% of total P intake but ranging up to 50%. The main source of P additives from single-ingredient food items was cola drinks, and the main food group sausage dishes. The intake of total P, but not P additives, was only weakly reflected in the U-P. Access to more comprehensive information on P additives in foods would improve further evaluation of potential health consequences.

## Author contributions

The authors’ responsibilities were as follows – AÅ: designed the research project; all authors: were involved in conducting the research; FS, JG, EP, AKL, SL, MK: provided essential materials; FS: analyzed data; FS, AÅ: wrote the article and had primary responsibility for final content; and all authors: read and approved the final manuscript.

## Conflict of interest

The authors report no conflicts of interest.

## Funding

This project was financially supported by the Swedish Research Council (Vetenskapsrådet; Grant Number: Dnr 2022-00980).

## Data availability

Data described in the manuscript, code book, and analytic code will be made available on request.
