# Supplementary material for: The Fate of Phosphate: Assessing Dietary Intake and Urinary Excretion in Swedish Adolescents
Source: Curr Dev Nutr. 2024 Jun 19;8(7):103799. doi: 10.1016/j.cdnut.2024.103799 (PMC11264180; doi:10.1016/j.cdnut.2024.103799)
Supplement: Multimedia component 1 [file mmc1.docx]

The fate of phosphate: Assessing dietary intake and urinary excretion in Swedish adolescents

Fredrik Söderlund, fredrik.soderlund@ki.se

**Table of contents**

[**Supplementary Table 1:** Industry data used for assessing Phosphate additives. 2](#_Toc166772436)

[**Supplementary Table 2:** Foods within the P database where total P was changed. 3](#_Toc166772437)

[**Supplementary Figure 1:** Sources of total P and their respective contributions to the intake of total P in the sub-population (n=756). 4](#_Toc166772438)

[**Supplementary Figure 2:** Sources of P additives and their respective contributions to the intake of P additives in the sub-population (n=756). 5](#_Toc166772439)

# **Supplementary Table 1:** Industry data used for assessing Phosphate additives.

| **Food category number** | **Food category name** | **Reported use level (mg/kg or mg/L P_2_O_5_)** | **Provider** |
| --- | --- | --- | --- |
| 01.4 | Flavoured fermented milk products including heat-treated products | 67 | EDA |
| 01.5 | Dehydrated milk | 733 | EDA |
| 01.7.5 | Processed cheese | 10535 | EDA |
| 01.8 | Dairy analogues | 456 | FDE |
| 3 | Edible ices | 113 | FDE |
| 04.2.6 | Processed potato products (meal) | 260 | FDE |
| 04.2.6 | Processed potato products (fried) | 1078 | FDE |
| 05.3 | Chewing gum | 4986 | ICGA |
| 06.5 | Noodles (noodle meals) | 96 | FDE |
| 06.6 | Batters | 5286 | FDE |
| 07.1 | Bread and rolls (final product) | 714 | FDE |
| 07.1 | Bread and rolls (only soda bread) | 3155 | FDE |
| 07.2 | Fine bakery wares | 2260,1 | FDE |
| 08.3.1 | Non heat-treated processed meat | 9 | FDE |
| 08.3.2 | Heat-treated processed meat | 725 | FDE |
| 09.1.2 | Unprocessed molluscs and crustaceans | 4000 | CEFIC^a^ |
| 12.5 | Soups and broths | 149 | FDE |
| 12.6 | Sauces | 445 | FDE |
| 13.3 | Dietary foods for weight control | 503 | SNE |
| 14.1.4 | Flavoured drinks | 223 | FDE |
| 14.1.4 | Flavoured beverages | 4000 | CEFIC^a^ |
| 14.1.5.2 | Other (instant tea) | 47 | FDE |
| 16 | Desserts | 917 | FDE |

CEFIC: European Chemical Industry Council; EDA: European Dairy Association; FDE: Food Drink Europe; ICGA: International Chewing Gum Association; SNE: Specialised Nutrition Europe

* For food categories with more than one provider of usage levels, we used values with the greatest number of samples classified as non-niche products, with the exception for chewing gum, where the only value available was for niche products.

^a^ As the European Chemical Industry Council (CEFIC) is an organization for chemical producers and suppliers, rather than a food producer, the values from CEFIC were only used when no other value was provided.

# **Supplementary Table 2:** Foods within the P database where total P was changed.

| **Food** | **Previous P (mg/100g)** | **New P value (mg/100g)** |
| --- | --- | --- |
| Mushroom sauce | 28 | 47 |
| Caesar salad chicken salad with dressing | 183 | 192 |
| Pie chicken bacon | 105 | 174 |
| Deep fried fish | 167 | 259 |
| Deep fried onion rings | 58 | 150 |
| Chili cheese | 161 | 253 |
| Muffins | 140 | 239 |
| Processed cheese (mjukost) | 767 | 1227 |
| Baguette sub med topping (chicken and cheese, cheese) | 194 | 263 |
| Sandwich subway (meat and cheese, chicken and cheese) | 142 | 211 |

P: phosphorus

# **Supplementary Figure 1:** Sources of total P and their respective contributions to the intake of total P in the sub-population (n=756).

# **Supplementary Figure 2:** Sources of P additives and their respective contributions to the intake of P additives in the sub-population (n=756).
